# Validating the Efficacy of a Mobile Digital Therapeutic for Insomnia (WELT-I): Randomized Controlled Decentralized Clinical Trial

**DOI:** 10.2196/70722

**Published:** 2025-09-23

**Authors:** Kyung Mee Park, Suonaa Lee, Yujin Lee, Daa Un Moon, Eun Lee

**Affiliations:** 1 Department of Hospital Medicine, Yongin Severance Hospital, Yongin, Republic of Korea; 2 Department of Psychiatry and Institute of Behavioral Science in Medicine, Yonsei University College of Medicine, 50-1 Yonsei-ro, Seodaemun-gu, Seoul, 03722, Republic of Korea, 82 2 2228 1620; 3 Department of Pharmacy, College of Pharmacy, Yonsei Institute of Pharmaceutical Sciences, Yonsei University, Incheon, Republic of Korea; 4 WELT Corp, Seoul, Republic of Korea; 5 Department of Psychiatry and Neurosciences, Charité Campus Mitte, Charité - Universitätsmedizin Berlin, Berlin, Germany; 6 Department of Consultation-Liaison Psychiatry and Psychosomatic Medicine, University Hospital Zurich, University of Zurich, Zurich, Switzerland; 7 Institute for Innovation in Digital Healthcare, Yonsei University, Seoul, Republic of Korea

**Keywords:** decentralized clinical trial, insomnia, behavioral sleep medicine, cognitive behavioral therapy, digital therapeutics, digital therapeutics for insomnia, digital intervention, mHealth, mobile health

## Abstract

**Background:**

Cognitive behavioral therapy for insomnia (CBT-I) has proven to be an effective treatment; however, its accessibility is limited. To address this issue, digital therapeutics for insomnia (DTx-Is), which are software-driven interventions designed to treat insomnia based on CBT-I, have emerged as a potential solution to enhance access.

**Objective:**

This study aimed to verify the efficacy and safety of WELT-I, a DTx-I. Due to the impact of the global pandemic during the study period, we thought that a decentralized clinical trial (DCT) design that does not require visits to institutions would be appropriate for a clinical study of a digital therapeutic for patients with insomnia. Thus, we also examined the potential of the DCT design as an effective method for validating DTx-Is.

**Methods:**

A double-blind, sham-controlled randomized DCT was conducted with participants who met the diagnostic criteria for insomnia. Participants were recruited through advertisements posted on an open-access website. WELT-I is a DTx-I based on CBT-I. A sham app was engineered to mirror WELT-I’s installation, login, user engagement, and content delivery processes while maintaining double-blind protocols. After randomization, participants were asked to use WELT-I or the sham app for 6 weeks. All treatment processes were fully automated. Sleep parameters were measured through an app-based sleep diary. Self-report questionnaires on sleep, depression, and anxiety were administered via the app at baseline and the end of the study. The primary outcome was sleep efficiency. To investigate the feasibility of the DCT design, compliance, retention rate, participant satisfaction, and time to reach the recruitment goal were evaluated.

**Results:**

A total of 89 participants provided consent and underwent screening, and 68 participants were randomly assigned to the WELT-I group (n=33) or control group (n=35). Among them, 14 participants discontinued the trial, leaving 54 participants who completed the study and were included in the final analysis (28 in the WELT-I group and 26 in the control group). WELT-I significantly improved sleep efficiency (least-squares difference=8.28; *P*=.04) and dysfunctional beliefs about sleep (least-squares difference=−1.03; *P*=.008) compared with the sham app. The study completed recruitment in 73 days, and the compliance rate was 95% (186/196) in the WELT-I group and 91% (165/182) in the control group. Moreover, the retention rate was 82% (23/28), and the average satisfaction score was 7.2 out of 10.

**Conclusions:**

WELT-I showed significant therapeutic efficacy and safety in improving sleep efficiency and sleep-related dysfunctional attitudes in cases of insomnia. In addition, this study demonstrated the feasibility of DCTs, and the findings of rapid recruitment, high compliance and retention rates, and strong participant satisfaction suggest that DCTs have sufficient potential to be expanded to clinical studies verifying the efficacy of other DTx-Is in the future.

## Introduction

Insomnia, one of the most prevalent health complaints, affects 30%‐35% of individuals annually, with 10% meeting the Diagnostic and Statistical Manual of Mental Disorders-IV criteria for clinical diagnosis [1-3]. It is associated with significant health risks, including increased mortality, metabolic syndrome, cardiovascular diseases, psychiatric disorders, and impaired neurobehavioral performance [4-13]. Cognitive behavioral therapy for insomnia (CBT-I), recommended as a first-line treatment, has proven effective, demonstrating long-term benefits and minimal side effects compared with hypnotic drugs [14-16]. However, barriers, such as limited insurance coverage, low reimbursement rates, a shortage of trained professionals, and the time-intensive nature of the therapy, hinder its accessibility [17-19].

To address these challenges, digital therapeutics for insomnia (DTx-Is), which represent a type of digitalized CBT-I, have been developed. Digital therapeutics are defined as evidence-based therapeutic interventions for patients, which are driven by high-quality software programs to prevent, manage, or treat a medical disorder or disease [20]. DTx-Is specifically deliver CBT-Is, which are standardized and effective treatments for insomnia. DTx-Is, exemplified by Somryst (Food and Drug Administration approved) and Sleepio (Conformité Européenne marking), could offer greater accessibility compared to traditional face-to-face CBT-I while maintaining therapeutic impact, enabling personalized care without the constraints of time and location [21]. The efficacy and safety of these DTx-Is (Somryst and Sleepio) for treating patients with insomnia have been proven in randomized controlled trials (RCTs) [22,23].

“WELT-I” is a Korean DTx-I developed by WELT Co, Ltd (Seoul, Republic of Korea). It is a fully digitalized, nonpharmacological treatment app. Similar to other DTx-Is, such as Somryst and Sleepio, WELT-I incorporates core CBT-I components, and it uses a built-in algorithm to provide users with personalized feedback and educational content related to insomnia. While Somryst is a prescription-only mobile app targeting chronic insomnia with comorbid depression [24] and Sleepio offers an English-language web- and mobile-based program guided by an animated virtual therapist and is accessible directly to consumers without a prescription [25], WELT-I distinguishes itself from these DTx-Is by providing content tailored specifically for Korean users and is available with a physician prescription. It integrates user-reported sleep data, offers physician-linked dashboards to support real-time clinical decision-making, and is designed for seamless incorporation into routine clinical practice in Korea.

Since traditional RCTs were used to confirm the effectiveness of all commercially available DTx-Is [22,23], a conventional RCT was initially planned to assess WELT-I, in which participants would visit a medical institution, interact face-to-face with researchers, and be prescribed either the DTx-I or a control app. RCTs involving face-to-face interaction are considered to provide the highest level of clinical evidence and are essential for establishing credibility, safety, and efficacy [26,27]. However, the global pandemic made it difficult for participants to travel to the trial site and engage in face-to-face communication, posing a significant challenge during the planning of the WELT-I trial. Moreover, 1 of the key advantages of digital therapeutics is their ability to reduce the burden on patients by eliminating the need for in-person visits to medical institutions for treatment [28,29]. However, requiring participants to visit medical institutions in order to participate in clinical trials may undermine the inherent benefits and real-world applicability of digital therapeutics.

Decentralized clinical trials (DCTs) are clinical trials in which some or all trial-related activities take place outside of traditional research sites, such as hospitals or clinics [30]. DCTs have gained attention as a viable solution to the limitations of conventional clinical trials, particularly during the COVID-19 pandemic. By enabling research activities to occur beyond the confines of the study site, DCTs improve participant accessibility and engagement throughout the trial process [31,32]. DCTs can increase the efficiency of recruitment of study subjects and encourage participation from more diverse populations. In addition, advances in digital health care technologies, such as electronic consent, electronic medical record systems, mobile apps, and wearable devices, have enabled continuous remote monitoring [33]. DCTs thus offer several potential benefits for participants, including reduced time and decreased financial and psychological burdens [34]. The approach is considered suitable for intervention trials that do not rely heavily on face-to-face interactions, such as mobile technologies, home health platforms, and virtual-first designs [35].

For these reasons, we considered a DCT to evaluate the efficacy and safety of WELT-I. Since WELT-I is an intervention that could be monitored via an app and the data are stored in the cloud via the app, the clinical study could be conducted without the participants visiting the site. This study aimed to (1) assess the efficacy and safety of WELT-I in patients with insomnia, and (2) examine the potential of the DCT design as an effective method for validating DTx-Is. To evaluate the usability and suitability of WELT-I, we designed a randomized, sham-controlled, double-blind study, and to evaluate the usability and suitability of the DCT protocol, we evaluated compliance, retention rate, participant satisfaction, and time to reach the recruitment goal.

## Methods

### Participants

The study participants were adults aged 19 to 65 years, who met the Diagnostic and Statistical Manual of Mental Disorders-5 criteria for insomnia disorder [36] and had an Insomnia Severity Index (ISI) score of 8 or higher [37]. All participants were required to demonstrate proficiency in using smartphone apps. After enrollment, the participants were asked to maintain a sleep diary for 1 week to assess their sleep patterns for screening purposes. Participants who showed low adherence by failing to complete the sleep diary daily and those with a sleep efficiency of 80% or higher were excluded from the study.

The exclusion criteria were as follows: diagnosis of sleep disorders other than insomnia (eg, sleep-related breathing disorders, parasomnias, restless legs syndrome, narcolepsy, or circadian rhythm disorders); concurrent nonpharmacological treatment for insomnia (eg, CBT-I, light therapy, or traditional sleep-related treatments); ongoing psychotherapy within the previous 3 months (eg, cognitive behavioral therapy, motivational enhancement therapy, or psychoanalysis); comorbid medical conditions, including active or progressive illnesses (eg, congestive heart failure, chronic obstructive pulmonary disease, or acute pain), neurological disorders (eg, cerebrovascular diseases), neurodegenerative diseases (eg, dementia or multiple sclerosis), and unstable medical conditions; a life expectancy of less than 6 months; any current or past major psychiatric disorders (eg, schizophrenia or related psychotic disorders, bipolar disorder, major depressive disorder, or substance use disorder); a history or risk of suicide (Columbia-Suicide Severity Rating Scale score of 4 or higher [38]); recent changes in the dosage of psychiatric medications, including sleep medications; risk of occupational accidents due to sleep deprivation; engagement in shift work; current pregnancy or a plan to become pregnant during the clinical trial period; any difficulty in understanding or communicating about the clinical trial as determined by the investigator; participation in another clinical trial within 4 weeks prior to screening; and difficulty completing virtual trial procedures.

### DCT Design

We conducted a double-blind, sham-controlled, randomized, parallel DCT to evaluate the efficacy and safety of a 6-week smartphone-based DTx-I compared with a self-developed sham app as a control. Participants were recruited through advertisements posted on an open-access website. Participants were able to see brief information about the purpose of the study, recruitment goals, exclusion criteria, the study participation period, study content including randomization, compliance requirements when participating in the study, expected benefits and risks, the sponsor, the research institution, etc, through an online poster. During the written consent procedure, participants were provided with detailed information about the study and contact information in case of safety- and security-related issues. Although we initially planned a fully remote decentralized trial, the institutional review board (IRB) requested an on-site visit for the informed consent process due to concerns related to participant identification. Therefore, participants visited the site in person during their initial visit to complete the e-consent process. After enrollment, all subsequent procedures were conducted remotely via the app or phone calls. The total study period was 7 weeks, including 1 week of screening.

At the baseline visit, enrolled participants were sequentially assigned a randomization number and allocated to the intervention or control group via an interactive web response system based on a pregenerated randomization sequence. Participants were allocated in a 1:1 ratio using a block randomization method stratified by baseline administration of sleep medication. The randomization sequence was generated by an unblinded statistician using Python (version 3.0 or higher) with the Numpy module (version 1.20.0 or higher). A combination of block sizes (eg, 4 and 2) was used to minimize predictability. To maintain allocation concealment, only the interactive web response system developer had access to the randomization list, and randomization details were unavailable to investigators and participants until the unblinding procedure following database lock. Both the participants and investigators remained unaware of the group assignments throughout the study. Participants were identified solely by their randomization numbers throughout the clinical trial, and randomization details were concealed until the unblinding procedure was complete. An unblinded research nurse facilitated app installation and monitored for adverse events but was not involved in data analysis or outcome assessments.

Each participant received a unique access code to download and log into the trial app (either WELT-I or the sham app) depending on their allocation. Participants accessed the app via their smartphones and were required to have a stable internet connection to use it effectively.

Throughout the trial, WELT-I and the sham app remained stable, with no significant technical disruptions. Minor bugs related to user interface inconsistencies in WELT-I were identified and resolved during the trial. These fixes were minor and did not affect the core functionality or content of the intervention. There were no downtimes or interruptions in app availability, and no changes were made to the content of either the intervention or comparator during the study period. Both the active and sham apps automatically expired 6 weeks after the start of the study.

**Figure 1.**
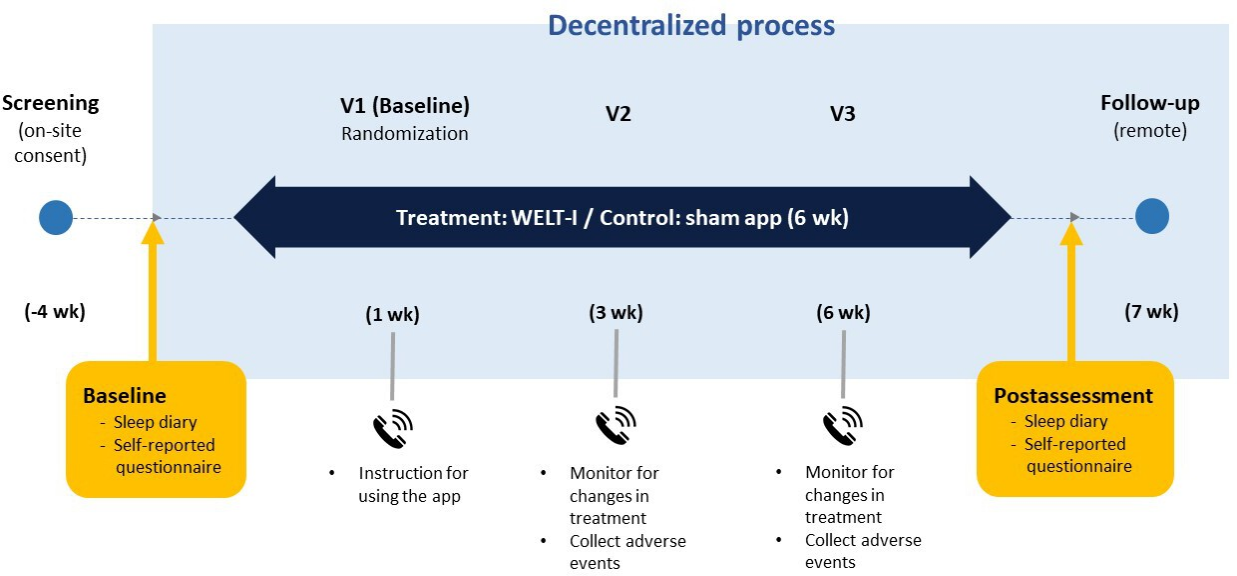
Schematic representation of the decentralized trial design.

Participants in the intervention group were encouraged to write a sleep diary throughout the treatment period, whereas participants in the control group were only asked to write a sleep diary for 7 days before starting the app and 7 days after the end of its use. Different sleep diary instructions for the control group were provided to minimize unintended effects, such as increased sleep-related cognition and awareness [39,40]. Both groups completed self-reported questionnaires at baseline and the end of the intervention and an app satisfaction assessment at the end of the study, through their apps. Telephone visits were performed in the first, third, and sixth weeks after baseline to monitor for any changes in concomitant drug usage and adverse events (Figure 1).

Due to protocol violations, 2 participants were excluded prior to randomization. Following randomization, 4 participants dropped out due to issues related to app installation or use, and 1 withdrew due to the use of prohibited medication. Three participants who had issues with delays in final visits after the treatment were still included in the final analysis. These deviations were monitored and appropriately reported to the IRB, and those affected by major deviations were either withdrawn or excluded from the per-protocol analysis set.

### DTx-I: WELT-I

WELT-I is a digital CBT-I available on both iOS and Android. The development of WELT-I adheres to good manufacturing practice standards, with certification obtained in June 2021. The program content of WELT-I includes 6 sessions of a multicomponent intervention covering sleep hygiene education, stimulus control, bedtime restriction, cognitive restructuring, and relaxation techniques. In the first session, users receive basic education on insomnia and relaxation training. The second session introduces sleep restriction and stimulus control principles, along with personalized relaxation content. The third session focuses on cognitive restructuring and sleep hygiene practices. The fourth session provides content on treatment completion and relapse prevention. In the fifth and sixth sessions, users continue to reinforce learned skills with additional educational content and tailored content recommendations based on prior inputs. A recommended bedtime schedule is generated based on the sleep diary data entered by the user into the app each day. A multidisciplinary team of experienced psychiatrists, software developers, product designers, and behavioral scientists collaborated to ensure the app’s clinical relevance, user accessibility, and adherence to evidence-based practice in CBT-I. Prior to the trial, we conducted a survey on the overall user experience of the app, including its functions and convenience, targeting patients with insomnia. We incorporated this feedback to improve the app and ensure it met the needs of the target population. Representative screenshots of the WELT-I app with English translations are presented in Multimedia Appendix 1.

Participants assigned to the WELT-I group were prescribed a sleep schedule via the app based on their sleep diaries from the previous 7 days, which they filled out on their own using the app. To enhance learning, the app content was unlocked weekly, 1 session at a time, based on the number of sleep diary entries and the passage of time.

### Sham App

As a control, a sham app was designed to replicate WELT-I in terms of installation, login, engagement, and content delivery, ensuring double-blindness. The purpose of using a sham app was to control for the potential therapeutic effects of app use itself, apart from the active components of the DTx-I, that could otherwise influence trial outcomes [41]. The sham app delivers sleep hygiene education covering the effects of sleep-disrupting substances like caffeine, nicotine, and alcohol; offers guidance on creating an optimal sleeping environment; and recommends foods that promote better sleep. Unlike the WELT-I app, all content in the sham app was accessible at the start of the intervention period, with no sequential unlocking.

### Outcome Variables

The primary outcome was change in sleep efficiency, a treatment target in traditional CBT-I [15]. Sleep efficiency was calculated by dividing total sleep time (TST) by time in bed and multiplying the result by 100. The secondary outcomes included changes in other sleep parameters, including TST, sleep onset latency (SOL), wake after sleep onset (WASO), number of awakenings (NOA), and sleep quality. TST was defined as the actual time spent sleeping (in minutes) while in bed. SOL was measured as the time taken to fall asleep (in minutes) after going to bed, and WASO was the total time (in minutes) spent awake from the initial sleep onset until the final awakening before getting out of bed. Sleep quality was assessed using a single-item questionnaire on a 5-point Likert scale, with higher scores indicating better sleep quality. All sleep-related data were collected via app-based sleep diaries. None of the participants used wearable sleep trackers for the study.

All participants completed the following self-reported questionnaires before and after the intervention: ISI, Dysfunctional Beliefs and Attitudes about Sleep-16 (DBAS-16), Patient Health Questionnaire-9 (PHQ-9), and Generalized Anxiety Disorder-7 (GAD-7). The scores obtained were used to measure changes in insomnia and other psychiatric symptoms. The ISI is a retrospective self-report questionnaire assessing the perceived severity of insomnia symptoms, with scores ranging from 0 to 28 and higher scores indicating greater severity [37,42]. Dysfunctional beliefs about sleep were assessed using the DBAS-16, with mean scores ranging from 1 to 10 and higher scores indicating a stronger endorsement of dysfunctional beliefs [43,44]. Depression was evaluated using the PHQ-9, with total scores ranging from 0 to 27 and higher scores indicating more severe depressive symptoms [45,46]. Anxiety was assessed using the GAD-7, with scores ranging from 0 to 21 and higher scores indicating greater anxiety symptom severity [47,48].

To investigate the feasibility of DCTs, we assessed compliance, retention rate, participant satisfaction, and time to reach the recruitment goal. Time to reach the recruitment goal was defined as the time between the enrollment of the first patient and the enrollment of the last patient. Compliance was defined as the completion rate of the sleep diary during the study period (the number of days completed out of the first 7 days). The retention rate was calculated as the proportion of participants in the WELT-I group who completed all lessons by the end of the intervention period. Participant satisfaction was assessed at the final visit using a 10-point Likert scale, where participants indicated their satisfaction and the likelihood of recommending the app to others. Adverse events occurring during the clinical trial period were also monitored.

### Statistical Analysis

The sample size was calculated using PASS 2020 [49] with a significance level of *α*=.05 and a statistical power of 1−*β*=0.9, targeting an effect size of 1.13 based on prior DTx-I research [22]. Accounting for an expected attrition rate of 30%, the initial recruitment goal was set at 26 participants per group.

The primary analysis followed the full analysis set principle under the intent-to-treat framework. Participants were analyzed based on their randomized group allocation, regardless of app usage. The full analysis set included participants who used the intervention or comparator at least once and provided at least one postbaseline sleep diary entry. Normality was assessed using Q-Q plots and the Shapiro-Wilk test. Continuous variables were compared between the 2 groups using independent *t* tests for normally distributed variables or the Mann-Whitney *U* test for nonnormally distributed variables. Categorical variables were compared using the chi-square test or Fisher exact test. Within-group changes were evaluated using paired *t* tests. To evaluate between-group differences in the primary and secondary outcomes of this study, an analysis of covariance controlling for age, sex, the baseline use of sleep medication, and the baseline value of each outcome variable was conducted. A safety set included all participants who had used the app at least once to identify adverse events during the intervention.

Effect sizes (Cohen *d*) were calculated to quantify the magnitude of observed differences. Missing data were addressed using the *last observation carried forward* method, incorporating data from scheduled and unscheduled visits within predefined visit windows. This approach was applied to account for participants who discontinued or had incomplete data during the trial. The significance threshold for all analyses was set at .05. All analyses were performed using SPSS Statistics software version 26.0 (IBM Corp) and R version 4.3.1 (RStudio Inc). Reporting was primarily conducted in accordance with the CONSORT-EHEALTH (Consolidated Standards of Reporting Trials of Electronic and Mobile Health Applications and Online Telehealth) recommendations [50]. The CONSORT (Consolidated Standards of Reporting Trials) checklist is included in Checklist 1.

### Ethical Considerations

Ethical approval was obtained from the IRB of Severance Hospital of Yonsei University Health System, Seoul, Korea (approval number: 1-2022-0054). All participants provided informed e-consent during on-site visits conducted by blinded investigators. The collected data were stored in a deidentified format. The study protocol has been registered at ClinicalTrials.gov (NCT05809544). Participants received compensation equivalent to US $70 at the end of their study participation.

## Results

### Participants

**Figure 2.**
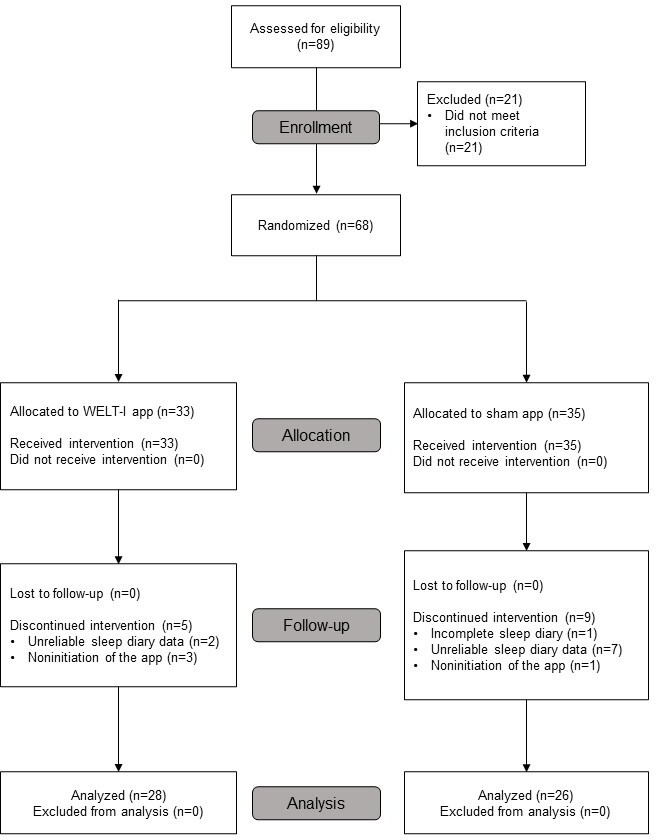
CONSORT (Consolidated Standards of Reporting Trials) flow diagram.

At the clinical trial site, 89 participants provided consent and underwent screening. As 21 participants did not meet the study criteria, they were excluded, and 68 participants were randomly assigned to the WELT-I group (n=33) or the control group (n=35). Of the 68 participants who were randomized, 14 discontinued the trial, and thus, 54 participants completed the study and were included in the final analysis (28 in the WELT-I group and 26 in the control group). The study period spanned from October 14, 2022, to February 20, 2023. There was no significant difference in the dropout rates between the 2 groups (*P*=.37) (Figure 2). The average age of the assigned participants was 38.7 years, and 74% (50/68) were women. There were no statistically significant differences between the 2 groups in terms of sex (*P*=.54), age (*P*=.44), marital status (*P*=.49), employment status (*P*=.43), alcohol use (*P*=.16), tobacco use (*P*=.14), or sleep medication use (*P*=.99) (Table 1).

**Table 1.** Demographic characteristics of the participants.

Variable		WELT-I (n=28)	Sham app (n=26)	*P *value
Age (years), mean (SD)		39.8 (12.2)	37.7 (12.6)	.54
Sex, n (%)				.44
Male		6 (21)	9 (35)	
Female		22 (79)	17 (65)	
Education, n (%)				.67
High school degree		4 (14)	6 (23)	
University degree		5 (18)	5 (19)	
Postgraduate degree		19 (68)	15 (58)	
Employment status, n (%)				.43
Employed		18 (64)	13 (50)	
Unemployed		10 (36)	13 (50)	
Marital status, n (%)				.49
Married		15 (54)	12 (46)	
Single		12 (43)	14 (54)	
Divorced		1 (4)	0 (0)	
Smoking status, n (%)				.14
Never		24 (86)	17 (65)	
Previous		1 (4)	5 (19)	
Current		3 (11)	4 (15)	
Alcohol intake, n (%)				.16
Never		13 (46)	9 (35)	
Previous		0 (0)	3 (12)	
Current		15 (54)	14 (54)	
Sleep medication, n (%)				.99
Taking		2 (7)	2 (8)	
Not taking		26 (93)	24 (92)	

### Treatment Effect of the DTx-I

The primary outcome, sleep efficiency, showed a statistically significant increase in both groups (both *P*<.001), though the improvement in the WELT-I group was significantly greater than that in the control group (*P*=.04). Dysfunctional beliefs about sleep also showed a greater improvement in the WELT-I group compared to the control group (*P*=.008). There were no significant differences between the 2 groups in other sleep parameters, including SOL (*P*=.23), WASO (*P*=.06), TST (*P*=.86), NOA (*P*=.69), sleep quality (*P*=.64), and insomnia severity (*P*=.24), despite significant improvements from baseline in the WELT-I group and the control group (SOL: *P*<.001 and *P*=.05, respectively; WASO: *P*<.001 and *P*=.05, respectively; TST: *P*=.03 and *P*=.01, respectively; NOA: *P*<.001 and *P*<.001, respectively; sleep quality: *P*<.001 and *P*<.001, respectively; insomnia severity: *P*<.001 and *P*<.001, respectively). The WELT-I group showed improvements in depression (*P*<.001) and anxiety (*P*=.05), but the differences between groups were not significant (depression: *P*=.15; anxiety: *P*=.40) (Table 2).

**Table 2.** The treatment effects of WELT-I and the sham app.

Parameter	WELT-I (n=28)		Sham app (n=26)		Least-squares difference		Cohen *d*, value (95% CI)	*P* value
	Baseline, mean (SD)	Posttreatment, mean (SD)	Baseline, mean (SD)	Posttreatment, mean (SD)	Mean (SD)	95% CI		
SE^a^	62.5 (11.9)	76.9 (14.8)	57.8 (12.5)	65.9 (16.0)	8.28 (3.96)	0.32 to 16.24	0.68 (0.13 to 1.23)	.04
SQ^b^	2.3 (0.4)	2.8 (0.6)	2.4 (0.5)	2.8 (0.6)	0.06 (0.14)	−0.21 to 0.34	0.14 (−0.40 to 0.67)	.64
SOL^c^	65.7 (43.2)	35.8 (61.0)	74.5 (60.1)	55.8 (43.6)	−16.03 (13.32)	−42.79 to 10.74	−0.31 (−0.85 to 0.23)	.23
WASO^d^	44.5 (36.7)	23.3 (24.6)	44.5 (36.9)	38.7 (38.0)	−15.84 (8.19)	−32.30 to 0.62	−0.43 (−0.97 to 0.11)	.06
TST^e^	320.1 (79.0)	363.9 (84.0)	290.4 (66.9)	342.2 (100.4)	4.17 (23.16)	−42.37 to 50.71	0.06 (−0.48 to 0.59)	.86
NOA^f^	2.2 (1.3)	1.2 (1.3)	1.9 (1.0)	1.2 (0.7)	0.10 (0.25)	−0.60 to 0.40	0.09 (−0.45 to 0.62)	.69
ISI^g^	16.6 (4.8)	10.4 (5.2)	17.2 (4.2)	12.2 (5.1)	−1.49 (1.25)	−4.01 to 1.02	−0.33 (−0.87 to 0.21)	.24
DBAS-16^h^	6.7 (1.2)	5.1 (2.0)	6.7 (1.2)	6.0 (1.2)	−1.03 (0.37)	−1.78 to −0.28	−0.88 (−1.44 to −0.33)	.008
PHQ-9^i^	9.9 (4.7)	5.2 (3.9)	8.3 (4.7)	6.2 (4.6)	−1.58 (1.09)	−3.76 to 0.60	−0.34 (−0.88 to 0.20)	.15
GAD-7^j^	6.1 (5.1)	4.1 (4.0)	4.9 (5.0)	4.7 (4.5)	−0.87 (1.04)	−2.96 to 1.21	−0.17 (−0.71 to 0.36)	.40

a SE: sleep efficiency (percentage).

b SQ: sleep quality (5-point Likert scale).

c SOL: sleep onset latency (minutes).

d WASO: wake after sleep onset (minutes).

e TST: total sleep time (minutes).

f NOA: number of awakenings.

g ISI: Insomnia Severity Index.

h DBAS-16: Dysfunctional Beliefs and Attitudes about Sleep Scale-16.

i PHQ-9: Patient Health Questionnaire-9.

j GAD-7: Generalized Anxiety Disorder-7.

### Feasibility of the DCT

The time to reach the recruitment goal of the DCT was 73 days (October 14 to December 26, 2022). The compliance rate was 95% (186/196) in the WELT-I group (n=28; 95% CI 0.91‐0.99) and 91% (165/182) in the control group (n=26; 95% CI 0.87‐0.96). The retention rate was 82% (23/28; 95% CI 0.68‐0.96). The mean participant satisfaction score was 7.21 (SD 2.08) on a 10-point scale, reflecting a generally favorable level of satisfaction and willingness to recommend the app to others.

### Adverse Events

The safety set included a total of 64 participants who had used the app at least once (30 in the WELT-I group and 34 in the control group). During the clinical trial period, adverse events occurred in 7 participants (11%; 8 cases). By group, adverse events were reported by 5 participants (17%; 6 cases) in the WELT-I group and 2 participants (6%; 2 cases) in the control group. The difference in adverse event incidence between the groups was not statistically significant (*P*=.24). All adverse events were mild in severity and were not considered related to the digital CBT-I intervention. In the WELT-I group, the reported adverse events included 2 cases of cystitis and 1 case each of nasopharyngitis, ligament sprain, abdominal discomfort, and asthma. In the control group, the reported events included 1 case of COVID-19 infection and 1 case of varicose veins. No moderate or severe adverse events were observed, and no adverse events required temporary discontinuation of the intervention. Additionally, no deaths or serious adverse events occurred during the study period.

## Discussion

### Principal Findings

We successfully validated the efficacy and safety of WELT-I through a double-blind, sham-controlled, randomized, parallel DCT. The results demonstrated that WELT-I significantly improved sleep efficiency and dysfunctional beliefs about sleep compared with the sham app. The findings of the time to recruit participants, compliance, retention rate, and participant satisfaction demonstrated the feasibility of this decentralized trial.

### Comparison With Prior Work

With a moderate effect size compared to the sham app, WELT-I showed improvement in sleep efficiency, which was chosen as the primary outcome of this study because it is a key behavioral target of CBT-I [15]. It is noteworthy that the app-based intervention alone, without therapist involvement, resulted in significant sleep efficiency improvements. Additionally, we observed a large effect size improvement in dysfunctional beliefs about sleep, with greater improvements in the WELT-I group compared with the control group, likely attributable to the cognitive therapeutic effect of WELT-I. The treatment effects of the WELT-I intervention align with findings from recent meta-analyses, supporting the efficacy of digital CBT-I [51-53]. The absence of a therapeutic effect of WELT-I on other sleep-related variables might be related to the improvements observed in both the WELT-I and control groups. Although there is no universal definition for a digital sham, it is generally described as a comparator that mimics a digital therapeutic’s design, components, and duration while removing or reducing the intensity of its active principle or component [41]. Therefore, in addition to the minimal therapeutic effects associated with some sleep hygiene content, the use of the app itself may have caused a placebo effect. By employing similar treatment delivery techniques as digital therapeutics, digital shams allow for double-blinding and may help reduce the risk of false-positive outcomes when assessing the effects of digital therapeutics.

Our study successfully completed participant recruitment within 73 days, using a DCT approach. This is a relatively short timeframe compared to similar DTx-I clinical trials, which required 6 to 24 months to recruit comparable sample sizes (25‐45 participants per group) [54-56]. This rapid recruitment aligns with trends observed in DCTs conducted in other clinical contexts. Another DCT study conducted in Korea, which was neither a DTx-I nor a DTx study, was able to complete recruitment of 30 subjects within 1 month, while a previous clinical study with a similar design required 12 months for recruitment [57]. Additionally, a review examining patient recruitment in DCTs reported that 11 of 13 studies achieved faster recruitment by applying the DCT design and using electronic recruitment methods compared with traditional clinical trials [58]. The results of this study align with the literature and support the possibility that the enhanced accessibility of DCTs encourages greater interest and participation in clinical trials.

The satisfactory levels of compliance, retention, and participant satisfaction observed in this study are also noteworthy results. Prior studies have suggested that a nonadherence rate of around 25% should be expected when implementing digital CBT [59,60]. In terms of decreased adherence due to limited face-to-face contact, the result appears to have been unaffected, possibly because the treatment intervention was based on digital therapeutics. Previous analyses of DCTs have indicated that using accessible digital content is associated with high retention rates, with 11 out of 13 studies in a review reporting strong retention [58]. Additionally, a previous study identified feedback options, reminders, and in-app coaching as key elements for promoting retention [61], all of which are also features of WELT-I. These findings suggest that future DCTs for digital therapeutics may also achieve high compliance and retention rates. Participant satisfaction with the digital content was also high in our study, indicating that the participants likely experienced little to no discomfort with the self-administered app and patient-centered trial design.

The findings from our study align with the widely recognized potential of DCTs to improve patient recruitment, compliance, retention, and engagement, allowing for continual data capture in real-world settings [30]. Previous studies on DCTs have also highlighted their potential benefits, such as eliminating travel burdens, enabling continual safety monitoring, and fostering patient engagement through digital platforms [30,62-64]. However, DCTs have also raised some ethical considerations, such as a shift in responsibilities toward participants’ trust and motivation, which are central to the researcher-participant relationship [27]. To mitigate these concerns, we implemented a single on-site visit during the early adoption phase of the DCT. However, according to the results of previous DCTs, e-consent can facilitate rapid recruitment of diverse participant populations in fully remote DCTs, suggesting a risk-benefit balance that future studies should consider [57,58]. Furthermore, DCTs may be less accessible for individuals with lower levels of digital literacy. Prior research has also described concerns regarding the exclusion of certain populations due to the use of apps and devices in trials [65,66]. In our study, the use of a digital therapeutic approach naturally attracted participants with some level of digital literacy, which partially mitigated this issue. However, as digital therapeutic trials expand to broader populations, including older adults with potentially lower digital literacy, researchers must consider how to address these accessibility issues.

### Strengths and Limitations

This study has several limitations. First, we did not conduct a more detailed analysis of retention rates and participant satisfaction. Tracking retention rates for each lesson would have provided a more comprehensive understanding of adherence, and assessing satisfaction for the app itself and the DCT environment separately could have allowed for a more focused evaluation of the DCT experience. Second, the sample size of the study was relatively small compared with previous clinical trials for digital CBT-I [67]. Although the number of participants in our study was sufficient for statistical validation, a larger sample size would have better leveraged the ability of the DCT to expedite the recruitment rate. Third, our study was not a fully remote DCT due to the single on-site visit requirement. Although the IRB’s recommendation to verify participants in person was ethically valid, even 1 on-site visit may have limited the study’s accessibility and impacted participant enrollment. Fourth, we did not use wearable sleep tracking devices in this study. These devices are widely used by general users due to their convenience in measuring sleep-related variables, and their adoption for research purposes is also increasing [68]. Compared to the current approach that relies on self-reported data, wearable devices offer the advantage of providing more objective sleep measurements. They can reduce feedback errors caused by missing or inaccurate user inputs, lower the burden on users, and enable more precise and user-friendly treatment delivery [69]. Given these strengths, wearable sleep trackers may serve as valuable tools to enhance both the measurement accuracy and therapeutic efficacy of DTx-Is. They may be particularly beneficial in DCTs, where minimizing in-person visits is essential. Lastly, our study was primarily designed to evaluate the clinical outcomes of the DTx-I, rather than to rigorously validate the DCT methodology itself. Our findings regarding the feasibility of a DCT are based on indirect measures, such as recruitment time, compliance, retention rate, and participant satisfaction, rather than a formal comparison with conventional RCT designs. This limits the generalizability of our conclusions about the applicability of DCTs in evaluating DTx-Is, and further studies are needed. Despite these limitations, this study is the first to attempt to validate DTx-Is through a DCT. The advantages of a DCT observed in this study were rapid participant recruitment, high compliance and retention rates, and high participant satisfaction. These findings suggest that a DCT may serve as an effective approach for not only validating DTx-Is but also assessing other digital therapeutics and conducting clinical trials that aim to minimize in-person contact. The results of this study provide a valuable basis for future DCTs.

### Conclusion

WELT-I showed significant therapeutic efficacy and safety in improving sleep efficiency and sleep-related dysfunctional attitudes in cases of insomnia. In addition, the findings of this study demonstrated the feasibility of DCTs, and the findings of rapid recruitment, high compliance and retention rates, and strong participant satisfaction suggest that DCTs have sufficient potential to be expanded to clinical studies verifying the efficacy of other DTx-Is in the future.

Given that digital therapeutics use software reflecting the latest technology, delays in recruitment and long trial durations can lead to the app being updated during the trial and the original software becoming outdated by the conclusion of the study [28]. In this context, DCTs may become increasingly valuable tools for measuring the clinical effectiveness of digital therapeutics, offering a timely and efficient clinical trial approach [70].

## Supplementary material

10.2196/70722 Multimedia Appendix 1 Representative screenshots of the WELT-I app.

10.2196/70722 Checklist 1 CONSORT-EHEALTH checklist (V 1.6.1).

## Abbreviations

CBT-I: cognitive behavioral therapy for insomnia
DBAS-16: Dysfunctional Beliefs and Attitudes about Sleep-16
DCT: decentralized clinical trial
DTx-I: digital therapeutic for insomnia
GAD-7: Generalized Anxiety Disorder-7
IRB: institutional review board
ISI: Insomnia Severity Index
NOA: number of awakenings
PHQ-9: Patient Health Questionnaire-9
RCT: randomized controlled trial
SOL: sleep onset latency
TST: total sleep time
WASO: wake after sleep onset
