# Supplementary figures and images for: Validating the Efficacy of a Mobile Digital Therapeutic for Insomnia (WELT-I): Randomized Controlled Decentralized Clinical Trial
Source: J Med Internet Res. 2025 Sep 23;27:e70722. doi: 10.2196/70722 (PMC12456842; doi:10.2196/70722)

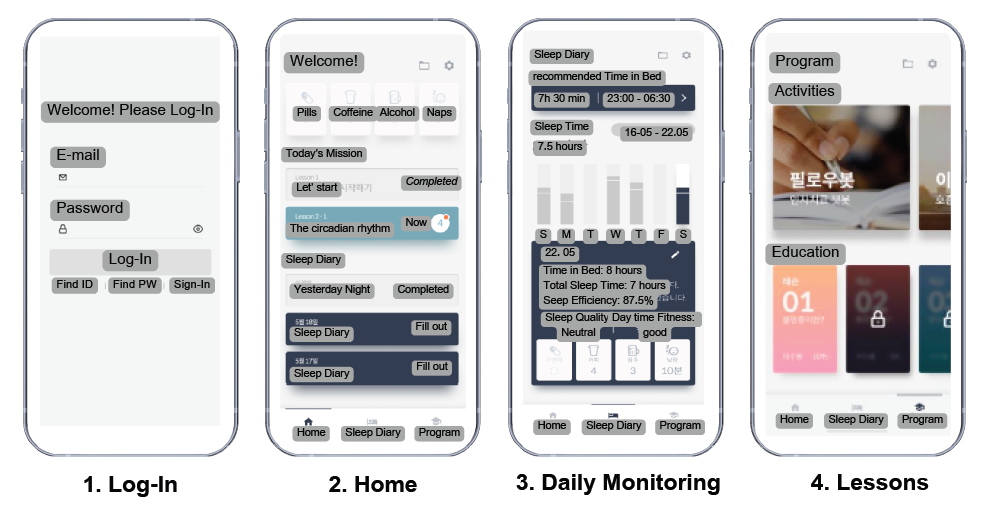

Supplement: Multimedia Appendix 1 [file jmir-v27-e70722-s001.PNG]
